# Zirconia Toughened Alumina Ceramics via Forming Intragranular Structure

**DOI:** 10.3390/ma17061309

**Published:** 2024-03-12

**Authors:** Junguo Li, Qiwang Cai, Guoqiang Luo, Xinyu Zhong, Qiang Shen, Rong Tu, Xiaoping Guo, Renchi Ding

**Affiliations:** 1 Chaozhou Branch of Chemistry and Chemical Engineering Guangdong Laboratory, Chaozhou 521000, Chinaqwcai@whut.edu.cn (Q.C.);; 2 Hubei Technology Innovation Center for Advanced Composites, Wuhan University of Technology, Wuhan 430070, China; 3 State Key Lab of Advanced Technology for Materials Synthesis and Processing, Wuhan University of Technology, Wuhan 430070, China

**Keywords:** intragranular structure, zirconia toughened alumina, transition alumina, mechanical properties

## Abstract

The distribution of second phase particles in the microstructure of composite ceramics affects the mechanical properties, and the intragranular structures often result in better properties compared to the intergranular structures. However, it is difficult to obtain composite ceramics with intragranular structure by conventional route. To produce composite ceramics with an intragranular structure in a simpler route. In this work, starting powders with different phase compositions were obtained by the co-precipitation method, and zirconia toughened alumina (ZTA) composite ceramics were prepared with these starting powders by spark plasma sintering (SPS). The results show that it is easier to fabricate ZTA composite ceramics with an intragranular structure by using composite powders containing amorphous or transition phase Al_2_O_3_ as starting materials. The phase composition of the powder prepared by the co-precipitation method after calcination at 1100 °C is θ-Al_2_O_3_ and t-ZrO_2_, and the average grain size after sintering at 1500 °C is 1.04 ± 0.28 µm, and the maximum Vickers hardness and fracture toughness of the specimens reach 19.37 ± 0.43 GPa and 6.18 ± 0.06 MPa·m^1/2^, respectively. The ZrO_2_ particles were the core of crystallization and grow together with the Al_2_O_3_ matrix, forming the intragranular structure of ZTA ceramics. This work may provide a new idea for preparing composite ceramics with intragranular structure.

## 1. Introduction 

Alumina (Al_2_O_3_) ceramics are widely used in a variety of advanced technologies due to its high hardness, strength, corrosion resistance and thermal shock resistance, however, the high brittleness restricts some of its applications [1,2]. In general, Al_2_O_3_-based composite ceramics possess better properties than single-phase Al_2_O_3_ ceramics [3,4,5,6]. Zirconia, especially stabilized t-ZrO_2_ (tetragonal zirconia), possess a high fracture toughness due to the phase transformation (tetragonal zirconia to monoclinic zirconia) toughening effect [7,8,9]. Ipek et al. [10]. added 10% zirconia to alumina, the Vickers hardness and fracture toughness of ceramics increased from 11.6 GPa and 3.2 MPa m^1/2^ to 16.8 GPa and 4.9 MPa m^1/2^, respectively. Therefore, composite ceramics of Al_2_O_3_ and ZrO_2_ exhibit both high hardness and high fracture toughness, commonly considered for the application of cutting tools [11,12], dies [13] and orthopedic implants [14], this composite ceramic material is known as ZTA (zirconia toughen alumina) ceramic. 

Sintering is the key process for the preparation of ZTA ceramics. The SPS technique enables rapid densification of nano powders through the application of pressure and high-intensity currents, making it a commonly employed method for the preparation of ZTA ceramics. It was known that SPS sintering enables high heating rate and short holding time thus lead to ceramics with a refined microstructure and resulting good mechanical properties [15]. Besides, the phase transformation assisted sintering is also an effective sintering technique [16].The phase transformation from metastable to stable phase easily occurs during the sintering process of starting powders with compositions of metastable phase, which promotes atomic diffusion and particle rearrangement, and facilitates efficient densification into a fully dense structure at lower temperatures without severe grain growth [17]. Eliko et al. [15] used porous γ-Al_2_O_3_ as the raw material and obtained alumina ceramics with a relative density of 99% by SPS sintering at 1550 °C, but applying pressure at high temperatures led to rapid grain growth with an average grain size of 8.5 µm. Xu et al. [16] prepared amorphous composite powders composed of 60 mol% alumina and 40 mol% stabilized zirconia using the sol-gel method, ZTA composite ceramics were obtained by hot pressing and the amorphous powders were used as starting powders. The results show that amorphous powders can be sintered into dense composite ceramics at much lower temperatures, and that the easier densification of amorphous powders is because of the higher free energy and finer particle size of their metastable states. Therefore, the phase composition of the starting powder has an important effect on the microstructure and mechanical properties of the ceramics. To better compare the effects of starting powders with different phase compositions on the microstructure and mechanical properties of ZTA ceramics, in this study, starting powders with different phase compositions were prepared using the co-precipitation method, which requires only a change in the calcination temperature to obtain powders with different phase compositions, and SPS was used to achieve rapid sintering of these powders.

The microstructure of the grains in composite ceramics is also an important factor affecting the mechanical properties. Previous research shows that preparing composite ceramics with intragranular structure could improve their mechanical properties. Niihara [18] classified nanocomposites into three types based on the position of SiC in the Al_2_O_3_ matrix: intragranular type (the ZrO_2_ grains are located in the interior of the Al_2_O_3_ grains), intergranular type (the ZrO_2_ grains are located in the boundaries of the Al_2_O_3_ grains) and intergranular/intergranular type. If most of the SiC was located inside the Al_2_O_3_ grains, the mechanical properties of the composites are significantly improved. Verma et al. [19] prepared zirconia reinforced alumina ceramics with intragranular structure by sintering powders obtained through sol-gel method, which improved hardness and wear resistance as compared to the conventional works. Du et al. [20] prepared ZTA ceramics with a large amount of intragranular structure by microwave sintering, with relative densities, flexural strengths, fracture toughness, and Vickers hardness of 99.07%, 520.21 MPa, 7.64 MPa·m^1/2^, and 18.79 GPa, respectively. However, there is no systematic discussion on the formation conditions of the composite ceramics with intragranular structure. The content of the second phase, the dispersion of the starting powder and the mixing uniformity of each phase may affect the formation of the intragranular structure. Therefore, it is difficult to prepare composite ceramics with a large amount of intragranular structure, the usual process for mixing the matrix powder and the second phase powder directly for sintering often results in composites with an intergranular structure. How to stably control the formation of a large amount of intragranular structure is a problem worth studying.

In this paper, a new and simple route is used to prepare ZTA ceramics containing a large number of intragranular structures, which does not require a powder mixing step: starting powders with different phase compositions were obtained at different calcination temperatures in the co-precipitation method, ZTA composite ceramics with intragranular structures were prepared by SPS using powders with different phase compositions. Amorphous and transition phase powders are much easier to the formation of intragranular structure in the SPS sintering of ZTA ceramics, the mechanical properties and formation mechanism were presented.

## 2. Materials and Experimental Methods

Al_2_O_3_-ZrO_2_ composite powders were prepared by co-precipitation method with composition of 85 wt.% Al_2_O_3_ and 15 wt.% ZrO_2_. Ammonium aluminum sulfate dodecahydrate (AlNH_4_(SO_4_)_2_·12H_2_O, AR, 99.0%), zirconyl chloride octahydrate (ZrOCl_2_·8H_2_O, AR, 99.0%) and ammonium bicarbonate (NH_4_HCO_3_, AR, 99.0%) were used as the raw materials, all these materials were provided by Innochem Technology, Beijing, China.

In powder production, the precipitant solution of NH_4_HCO_3_ was first prepared by dissolving in deionized water, the metal salt solution of AlNH_4_(SO_4_)_2_·12H_2_O and ZrOCl_2_·8H_2_O were also prepared by dissolving in deionized water and stirred for 60 min using a stirrer. Then, the metal salt solution was added drop wise into the constantly stirred precipitant solution for precipitation at 45 °C. At the end of precipitation, the solution was stirred for an additional hour to ensure full reaction completion. After the reaction, the precipitate was aged in the original solution for 24 h and washed three times with deionized water to remove impurity ions and then washed twice with ethanol to replace the deionized water. Finally, the ethanol washed precipitate was calcinated at 700–1300 °C in a muffle furnace for 2 h after dried at 60 °C for 24 h. After calcination, the powders were compacted by SPS to 10 mm diameter discs at 1500 °C for 10 min with heating rate of 100 °C/min and axial pressure of 50 MPa.

## 3. Characterization Methods

Phase analysis of powders and sintered samples were performed by Rigaku instrument by using Cu Kα radiation X Ray diffraction (XRD, SmartLab, Rigaku, Tokyo, Japan), in the 2θ range of 10–80°. To determine the temperature of thermal decomposition and phase transformations of powders in calcination. Thermal Gravimetry (TG) and Differential Scanning Calorimetry (DSC, Mettler Toledo, Zurich, Switzerland) measurements of dried powders were performed in a temperature range of 40–1400 °C at a scanning rate of 20 K/min in N_2_ (nitrogen).

The morphology and particle size of powders were examined by scanning electron microscope (SEM, Regulus8100, Hitachi, Tokyo, Japan). In order to observe the surface and microstructure of sintered samples, the surface of each sample was grounded and polished through polishing machine using a diamond paste and put in a box furnace for heat treatment at 1300 °C for 30 min. The microstructure was observed through SEM. Based on the SEM images, the grain size of sintered samples was measured by linear intercept method in Nano Measurer software (Version 1.2), and 100 grains were measured for each sample. The density of all sintered samples was evaluated by Archimedes method. The Vickers hardness of the polished samples was evaluated by a hardness tester (430SVD, Wolpert, Norwood, MA, USA) under a load of 49 N for 15 s with 10 points, and calculated by the follow formula [21]:

(1) $$H=0.18544\left(\frac{P}{d^{2}}\right)$$

where, *P* is the applied load, and *d* is the average length of the two diagonal lines of the indentation.

The fracture toughness of sintered samples was examined by indentation method (IM) and calculated by the follow formula [22]:

(2) $$K_{IC}=0.016{\left(\frac{E}{H}\right)}^{1/2}(\frac{P}{c^{3/2}})$$

In this formula, *K_IC_* is fracture toughness, *E* is Young’s Modulus (348 GPa in this work, obtained by measuring the sound velocity of the samples), *H* is Vickers hardness, *c* is half length of crack, *P* is the load. 

## 4. Results and Discussion

### 4.1. Powder Preparation

**Figure 1:**
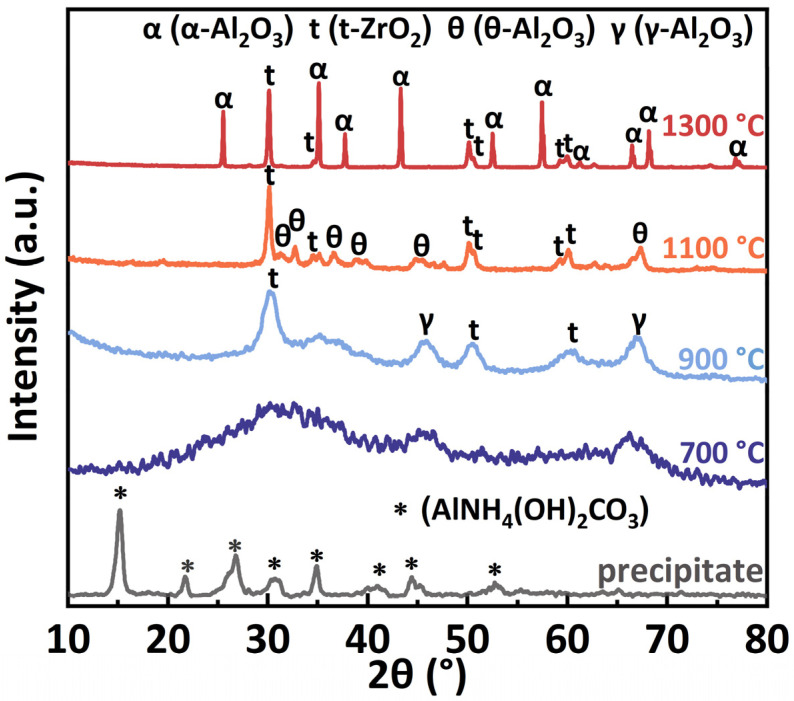
XRD patterns of the precipitate and ZTA powders calcinated at different temperature.

Figure 1 shows the XRD patterns of the precipitate and ZTA powders calcinated at different temperature, the phase evolutions are presented. For the precipitate, only X-ray peaks of ammonium aluminum carbonate hydroxide (AlNH_4_(OH)_2_CO_3_, AACH) were present, indicating that the components containing zirconium were amorphous cause the Zirconium hydroxide (Zr(OH)_4_) precipitates were amorphous. After calcinated at 700 °C, the XRD pattern was composed of only broad maxima with no obvious peaks, indicating that both Al_2_O_3_ and ZrO_2_ were amorphous. Diffraction of γ-Al_2_O_3_ and t-ZrO_2_ appeared when the calcination temperature raised to 900 °C because of the crystallization from the amorphous powders. The phase transformation from γ-Al_2_O_3_ to θ-Al_2_O_3_ took place at the calcination temperature of 1100 °C. The diffraction peaks of α-Al_2_O_3_ were detected in the powders after calcinated at 1300 °C because of the phase transformation from θ-Al_2_O_3_ to α-Al_2_O_3_. Due to the stabilization of Y_2_O_3_, ZrO_2_ has only tetragonal form and the diffraction peaks of m-ZrO_2_ (monoclinic zirconia) were not found at all calcination temperature.

**Figure 2:**
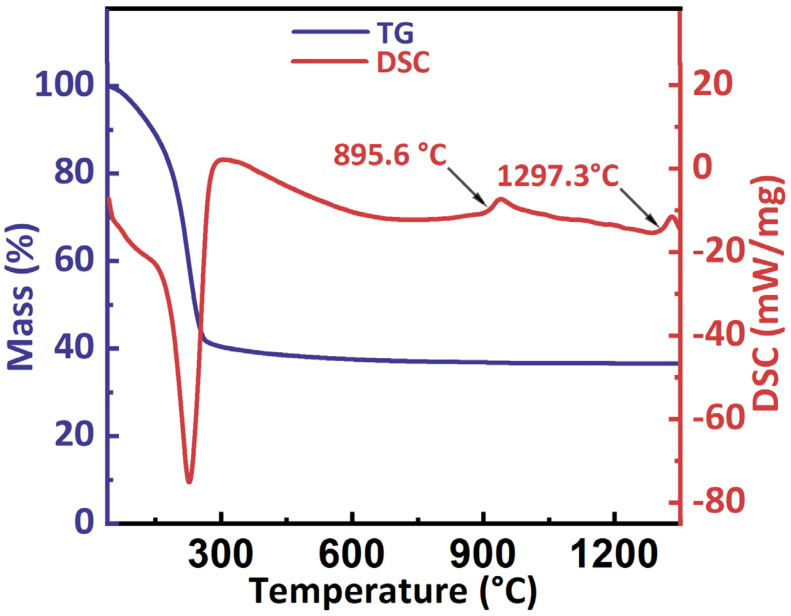
TG-DSC curves of the precipitate powders.

The TG-DSC curves of the precipitate are displayed in Figure 2. There was a large endothermic peak at about 227 °C and showed a large weight loss on the TG curve due to the decomposition of carbonate and hydroxide in the precipitate. There were two small exothermic peaks at 895.6 °C and 1297.3 °C was detected on the DSC curves. Based on the previous work on the phase transformation process of amorphous ZTA powders and the DSC curves of γ-Al_2_O_3_ [23,24], the peaks at 895.6 °C and 1297.3 °C on the DSC curves in this study could be attributed to the phase transformation of amorphous phase to γ-Al_2_O_3_ and θ-Al_2_O_3_ to α-Al_2_O_3_, respectively. Since the γ to θ phase transformation of Al_2_O_3_ is a non-reconstructive transition, there is no significant DSC signal detected. The temperature of the phase transformation to α-Al_2_O_3_ is about 1200 °C in pure Al_2_O_3_, which is lower than this work, indicating that the existence of ZrO_2_ hinders the phase transformation to α-Al_2_O_3._ In fact, it can be considered that the transformation to α-Al_2_O_3_ is inhibited by ZrO_2_ based on the work and conclusions of Ipek et al. [10] who synthesized Al_2_O_3_-ZrO_2_ composite powder by a co-precipitation method. Therefore, to study the influence of powders with different phase of Al_2_O_3_ on mechanical properties of ZTA ceramics through SPS sintering, the powders with calcination temperatures of 700 °C, 900 °C, 1100 °C and 1300 °C were selected as the starting powders for SPS sintering. The phase composition of the starting powders is listed in Table 1.

**Figure 3:**
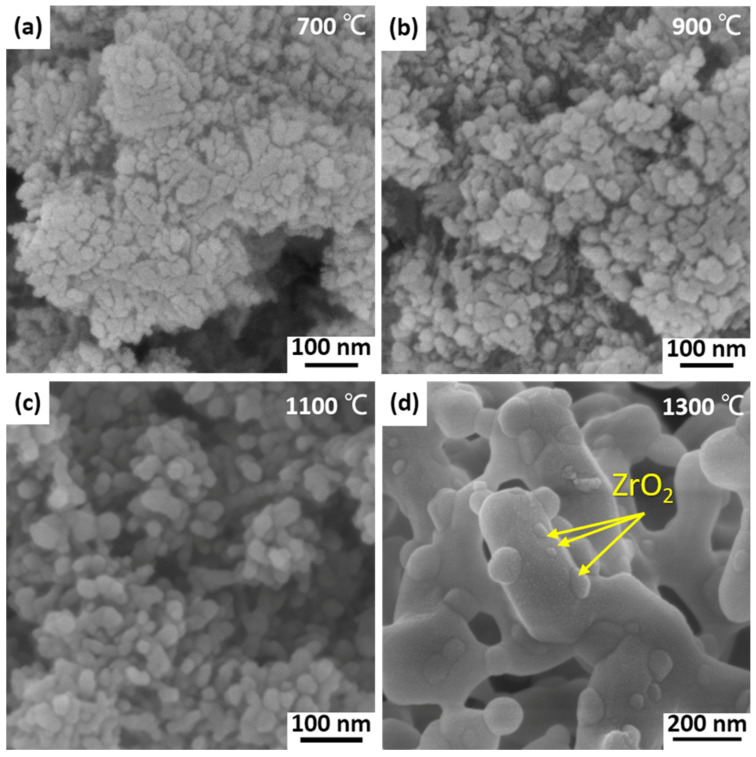
SEM images of (**a**) C700, (**b**) C900, (**c**) C1100, and (**d**) C1300 powders.

Figure 3 shows the SEM images of ZTA powders calcinated at different temperature. According to these images, the powders with the calcination temperature of 700 °C (C700 powders) was composed of extremely small particles, and there was no obvious boundary between the particles. The powders still agglomerated and the size has increased at the calcination temperature of 900 °C. When the calcination temperature rises to 1100 °C, the powder presents a better spherical shape and good dispersion with the size of about 50 nm. After calcination at 1300 °C, the Al_2_O_3_ particles grow rapidly and presented worm-like morphology with a larger size of about 500 nm. It is clear that smaller spherical ZrO_2_ particles are embedded on the surface and within the larger worm-like Al_2_O_3_ in Figure 3d. During the powder calcination stage before sintering, large amount of ZrO_2_, as the second phase, has been enveloped by large Al_2_O_3_ particles, forming an internal crystalline phase.

### 4.2. Microstructure of the ZTA Ceramics

**Figure 4:**
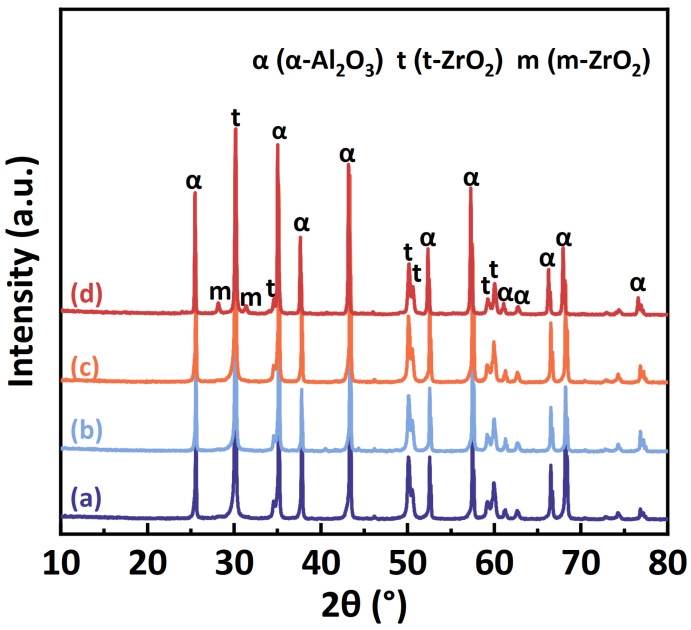
XRD patterns of samples sintered from (a) C700, (b) C900, (c) C1100, and (d) C1300 powders.

XRD patterns of samples sintered at 1500 °C are shown in Figure 4. Samples sintered from C700, C900 and C1100 powders consist solely of t-ZrO_2_ and α-Al_2_O_3_, whereas the sample sintered from C1300 powder contains a small quantity of m-ZrO_2_, this indicates that the Al_2_O_3_ matrix in the samples sintered from C1300 powders does not effectively bind the ZrO_2_ grains, and part of the t-ZrO_2_ to m-ZrO_2_ phase transformation occurred during the cooling process, resulting in the formation of m-ZrO_2_. Smuk et al. [25] reported that the t-ZrO_2_ is an important factor in improving the fracture toughness of ZTA composite ceramics. The transformation from the t-ZrO_2_ to m-ZrO_2_ absorbs the energy required for crack propagation, causes volume change, and inhibits crack propagation, increasing the fracture toughness of ZTA composite ceramics. 

**Figure 5:**
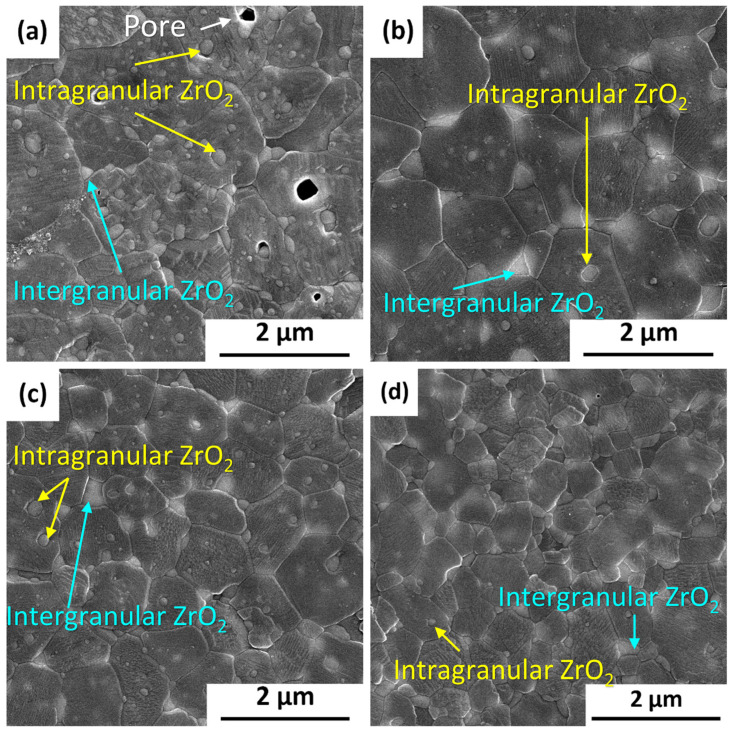
SEM micrographs of the polished surface of samples sintered from (**a**) C700, (**b**) C900, (**c**) C1100, and (**d**) C1300 powders.

**Figure 6:**
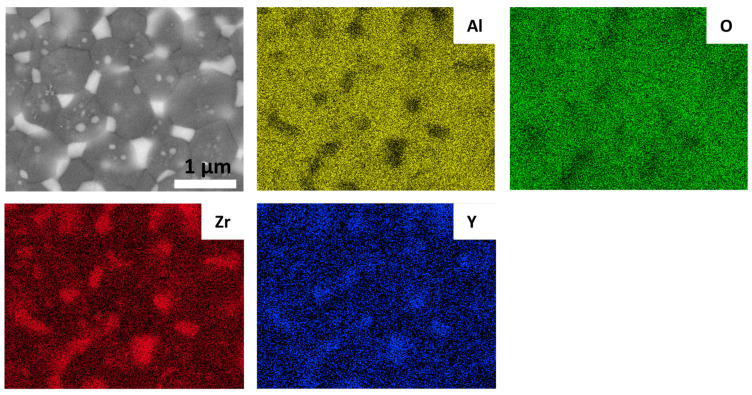
EDS mapping images for Al, O, Zr, Y elements of sample sintered from C1100 powders.

The microstructure of sintered samples was depicted in Figure 5, and the EDS mapping images was shown in Figure 6. According to the SEM and EDS images, the light regions belong to the phase enriched in Zr and Y while the dark phase were Al_2_O_3_ grains. In the samples sintered from C700, C900 and C1100 powders, the ZrO_2_ grains present two distribution states: intragranular type (the ZrO_2_ grains are located in the interior of the Al_2_O_3_ grains) and intergranular type (the ZrO_2_ grains are located in the boundaries of the Al_2_O_3_ grains). However, less amount of intragranular type ZrO_2_ was found in the samples sintered form C1300 powders, which indicates that the intragranular structure is more easily formed by SPS sintering of powder before the transformation to α-Al_2_O_3_ and the growth of the Al_2_O_3_. According to Niihara [18], the second phase particles are the core of crystallization, and the matrix and the second phase particles grow together to form the internal intragranular structure. In this work, zirconia particles can be considered as the crystal nucleus of Al_2_O_3_ phase transformation and crystallization. There is no systematic study on the formation mechanism of intragranular structures in composite ceramics, but it seems possible to fabricate composite ceramics with an intragranular structures by controlling the phase composition of the Al_2_O_3_ in the starting composite powder.

**Figure 7:**
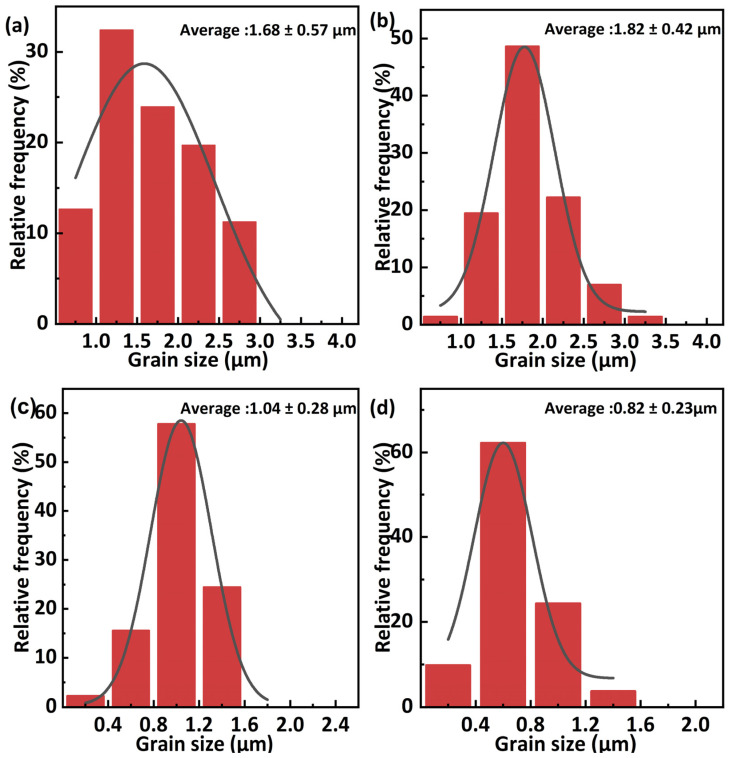
Al_2_O_3_ grain size distribution of samples sintered from (**a**) C700, (**b**) C900, (**c**) C1100, and (**d**) C1300 powders.

Figure 7 illustrates the distribution of Al_2_O_3_ grain sizes in sintered samples. The grain size initially increases and then decreases as the starting powder calcination temperature increases. The average grain sizes of Al_2_O_3_ in the samples were 1.68 ± 0.57 µm, 1.82 ± 0.42 µm, 1.04 ± 0.28 µm, and 0.82 ± 0.23 µm, correspondingly. This indicates that the grain size after sintering of amorphous and transition phase powders is much larger than that after sintering of crystalline powders. According to the research of Lee et al. [26], due to high driving force and the existence of singular boundaries of sintered body, amorphous alumina powders show abnormal grain growth after SPS sintering above 1500 °C and the number of abnormally grown grains increases with the duration of holding time, which does not occur in SPS sintering of crystalline powders. The metastable powders typically have higher sintering activity [27], in his work, C700, C900 and C1100 powders contains metastable Al_2_O_3_, which prone to the increasing of grain size during the sintering process.

### 4.3. Mechanical Properties of the ZTA Ceramics

**Figure 8:**
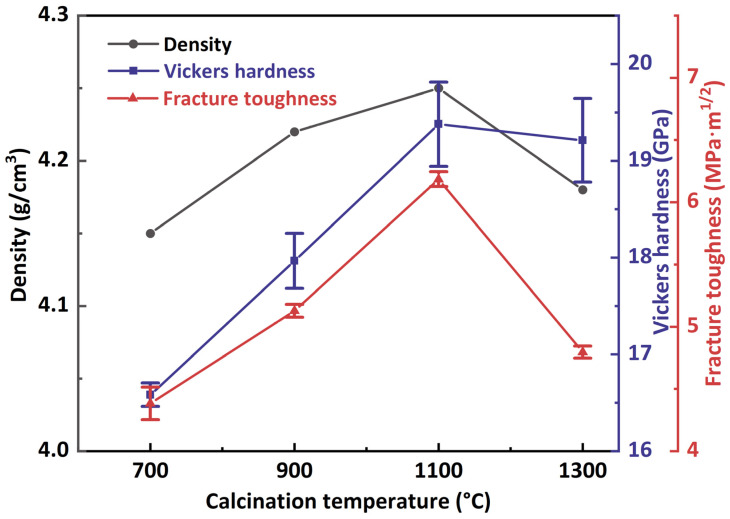
Influence of different calcination temperature of starting powders on relative density, hardness and fracture toughness after SPS sintering.

Figure 8 demonstrates influence of different calcination temperature of starting powders on relative density, hardness and fracture toughness after SPS sintering. The relative densities of the sintered samples first increased and then decreased with the increase of calcination temperature. The sample sintered from C700 powders shows low densities of 4.14 g/cm^3^ due to the fact that the powders were amorphous with very small particle size and need to undergo crystallization (amorphous → γ-Al_2_O_3_ and t-ZrO_2_) and phase transformations (γ-Al_2_O_3_ → θ-Al_2_O_3_ → α-Al_2_O_3_) in a very short sintering time, so the pores cannot be eliminated. Meanwhile, the severe agglomeration of the starting powders also makes it difficult to achieve densification. Due to the small particle size (50 nm) and good dispersion of the starting powders, the sample sintered form C1100 powders was densified with a higher relative density of 4.25 g/cm^3^. Because of the worm-like morphology and lager particle size of the powders with calcination temperature of 1300 °C, it is easier to form pores in sintering process, showing a lower relative density of 4.18 g/cm^3^, such pores are difficult to remove even after sintering at higher temperatures. The Vickers hardness and fracture toughness of the samples initially rise and then decline as the calcination temperature increases. The samples sintered from C700 and C900 powders exhibit lower hardness because of their bigger grain size and low densities. The sample sintered form C1100 powders displays significantly higher Vickers hardness of 19.37 ± 0.43 GPa and fracture toughness of 6.18 ± 0.06 MPa·m^1/2^. In comparison, the sample sintered from C1300 powders shows the Vickers hardness of 19.21 ± 0.44 GPa and extremely low fracture toughness of 4.79 ± 0.05 MPa·m^1/2^. This is due to fact that part of the t-ZrO_2_ in the sample cannot achieve effect of phase transformation toughening, resulting in lower fracture toughness than other samples. First, the intragranular structure can better bind the Al_2_O_3_ matrix to the ZrO_2_ particles and keep ZrO_2_ particles in the tetragonal phase, and the intragranular ZrO_2_ particles inhibit crack expansion, making the expansion path more tortuous and consuming a large amount of fracture energy to achieve the toughening effect. Second, the nano-composite effect could improve the hardness and fracture toughness of the material. Du et al. [20] supposed that the toughening mechanism of intragranular structure mainly results from local stresses surrounding the intragranular organization, which strengthens the primary grain boundaries and induces perforation fracture. Additionally, there is crack deflection, bridging, and the intragranular ZrO_2_ particles’ pulling out during the fracture process, as well as the t-ZrO_2_ to m-ZrO_2_ phase transformation contribute to the toughening effect [28]. Meanwhile, the presence of the intragranular structure leads to the appearance of sub grain boundaries within the Al_2_O_3_ grain, which is similar to the refinement of the structure and achieves the effect of fine-grained strengthening.

To better compare the mechanical properties of ZTA ceramics, the information from previous studies was summarized in Table 2. The fracture toughness values mostly ranged from 4 to 7 MPa·m^1/2^, while the Vickers hardness values ranged from 17 to 20 GPa. Obviously, ZTA ceramics with intragranular structure typically exhibit higher fracture toughness, indicating that intragranular structure effectively improved the fracture toughness of ZTA ceramics, but its relationship with hardness was not obvious. Noted that Vickers hardness and fracture toughness are affected by multiple factors, and that the highest hardness and fracture toughness values can be achieved through a combination of fine grains, high relative densities and specific microstructures [29]. The C1100 powders (starting powders with a phase composition of θ-Al_2_O_3_ and t-ZrO_2_) possess the best mechanical properties after sintering due to the small particle size, good dispersion, and proper sintering activity. θ-Al_2_O_3_ as a transition Al_2_O_3_ contributes to the formation of the intragranular structure, resulting in relatively high Vickers hardness and fracture toughness of ceramics. Thus, the starting powders with a phase composition of θ-Al_2_O_3_ and t-ZrO_2_ possess relatively high Vickers hardness and fracture toughness after sintering due to its small particle size, good dispersion and suitable sintering activity.

The equations for calculating fracture toughness as follow [34,36]:

(3) $$K_{IC}=0.0309\times {\left(\frac{E}{H}\right)}^{0.4}\times \frac{P}{c^{1.5}}$$

(4) $$K_{IC}=0.16H\sqrt{a}{\left(\frac{c}{a}\right)}^{-\frac{3}{2}},for\left(\frac{c}{a}\right)>1.8$$

where, a is half of the indentation diagonal.

## 5. Conclusions

In this work, a new and simple route was employed to produce ZTA ceramics with intragranular structures. Al_2_O_3_–ZrO_2_ composite powders with different phase compositions were fabricated by controlling the calcination temperature using the co-precipitation method and the phase evolutions in calcination process are given. The effect of starting powders with different phase compositions on mechanical properties and microstructure of ZTA ceramics were investigated. The phase composition of the starting powders has an important influence on the microstructure and mechanical properties of ZTA composite ceramics. Using composite powders that contain amorphous or transition phase Al_2_O_3_ as starting materials makes it easier to fabricate ZTA composite ceramics with an intragranular structure but the grain size is larger compared to sintering the crystalline powder. Starting powders with a phase composition of θ-Al_2_O_3_ and t-ZrO_2_ sintered by SPS achieved hardness and fracture toughness of 19.37 ± 0.43 GPa and 6.18 ± 0.06 MPa·m^1/2^, respectively. The ZrO_2_ particles were the core of crystallization and grow together with the Al_2_O_3_ matrix, forming the intragranular structure of ZTA ceramics. This work could provide new guidance for fabricating intragranular structure composite ceramics.

## Figures and Tables

**Table 1:** Phase composition of the starting powders.

Sample Identity	Calcination Temperature (°C)	Phase Composition
C700	700	amorphous
C900	900	t-ZrO_2_ and γ-Al_2_O_3_
C1100	1100	t-ZrO_2_ and θ-Al_2_O_3_
C1300	1300	t-ZrO_2_ and α-Al_2_O_3_

**Table 2:** The mechanical properties of ZTA ceramics and related comparisons.

Material	ZrO_2_ Content	*K_IC_* (MPa·m^1/2^)	Equation Type of IM	Hv (GPa)	ZrO_2_ Distribution	Sintering
This work	25.0 wt.%	6.18 ± 0.06 (IM)	Anstis, Equation (2)	19.30 ± 0.43	Intragranular/intergranular	SPS
Al_2_O_3_/ZrO_2_ [30]	15.0 wt.%	6.41 (IM)	Anstis, Equation (2)	N/A	Intragranular/intergranular	SPS
Al_2_O_3_/ZrO_2_ [31]	34.9 wt.%	6.72 ± 0.30 (SENB)	N/A	17.21 ± 0.25	Intragranular/intergranular	HP
Al_2_O_3_/ZrO_2_ [32]	17.0 wt.%	5.7 (IM)	Niihara, Equation (3)	18.26	Intragranular/intergranular	HP
Al_2_O_3_/ZrO_2_/Cr_2_O_3_ [33]	15.0 wt.%	4.67 ± 0.03 (IM)	Anstis, Equation (2)	18.10 ± 0.15	Intergranular	PS
Al_2_O_3_/ZrO_2_ [10]	10.0 wt.%	4.90 ± 0.10 (IM)	Anstis, Equation (2)	16.80 ± 0.40	Intergranular	PS
Al_2_O_3_/ZrO_2_ [34]	20.0 wt.%	5.20 (IM)	Evans and Charles, Equation (4)	17.60	Intergranular	3D printing
Al_2_O_3_/ZrO_2_ [35]	12.5 wt.%	5.40 ± 0.40 (IM)	Anstis, Equation (2)	18.30 ± 0.40	Intergranular	PS

IM: indentation method, SENB: single-edge notched beam method, SPS: spark plasma sintering, HP: hot press, PS: pressureless sintering.

## Data Availability

All data, models, and code generated or used during the study appear in the submitted article.
